# Complete Genome Sequence of a Multidrug-Resistant *Salmonella enterica* Serovar Typhimurium var. 5− Strain Isolated from Chicken Breast

**DOI:** 10.1128/genomeA.01068-13

**Published:** 2013-12-19

**Authors:** Maria Hoffmann, Tim Muruvanda, Marc W. Allard, Jonas Korlach, Richard J. Roberts, Ruth Timme, Justin Payne, Patrick F. McDermott, Peter Evans, Jianghong Meng, Eric W. Brown, Shaohua Zhao

**Affiliations:** Division of Animal and Food Microbiology, Office of Research, Center for Veterinary Medicine, U.S. Food and Drug Administration, Laurel, Maryland, USAa; Department of Nutrition & Food Science and Joint Institute for Food Safety & Applied Nutrition, University of Maryland, College Park, Maryland, USAb; Division of Microbiology, Office of Regulatory Science, Center for Food Safety and Nutrition, U.S. Food and Drug Administration, College Park, Maryland, USAc; Pacific Biosciences, Menlo Park, California, USAd; New England BioLabs, Inc., Ipswich, Massachusetts, USAe

## Abstract

*Salmonella enterica* subsp. *enterica* serovar Typhimurium is a leading cause of salmonellosis. Here, we report a closed genome sequence, including sequences of 3 plasmids, of *Salmonella* serovar Typhimurium var. 5− CFSAN001921 (National Antimicrobial Resistance Monitoring System [NARMS] strain ID N30688), which was isolated from chicken breast meat and shows resistance to 10 different antimicrobials. Whole-genome and plasmid sequence analyses of this isolate will help enhance our understanding of this pathogenic multidrug-resistant serovar.

## GENOME ANNOUNCEMENT

*Salmonella enterica* is recognized as one of the most common bacterial causes of food-borne illness worldwide (1) and causes approximately 1.0 million cases in humans, resulting in 19,336 hospitalizations and more than 378 deaths each year in the United States (2). *Salmonella enterica* subsp. *enterica* serovar Typhimurium was the first and second most commonly isolated serovar from retail meats and humans, respectively, in 2011 (3, 4). *Salmonella* serovar Typhimurium is associated with invasive diseases, and it is the most common serovar associated with *Salmonella*-related deaths in the United States (5, 6). The National Antimicrobial Resistance Monitoring System (NARMS), which is responsible for monitoring antimicrobial resistance in *Salmonella*, reported in 2010 that *Salmonella* serovar Typhimurium was the predominant serovar (44% of isolates from humans and 81% of isolates from chicken breast) among all isolates that were resistant to ≥3 antimicrobial classes (7).

In this report, we announce the availability of a fully closed genome sequence, including sequences of three plasmids, of *Salmonella* serovar Typhimurium strain CFSAN001921, which was isolated from chicken breast in New York in 2011. Pulsed-field gel electrophoresis (PFGE) showed that the isolate CFSAN001921 has the XbaI pattern JPXX01.1836. The isolate is resistant to 10 antimicrobials, including ceftriaxone, amoxicillin-clavulanic acid, ampicillin, cefoxitin, kanamycin, streptomycin, tetracycline, sulfamethoxazole, ceftiofur, and trimethoprim-sulfamethoxazole.

The genomic DNA of strain CFSAN001921 was isolated from overnight cultures using the DNeasy blood & tissue kit (Qiagen Inc., Valencia, CA) and sequenced using the Pacific Biosciences (PacBio) *RS* sequencing platform. Specifically, we prepared a single 10-kb library that was sequenced using C_2_ chemistry on 8 single-molecule real-time (SMRT) cells with a 90-min collection protocol on the PacBio *RS*. The 10-kb continuous-long-read (CLR) data were *de novo* assembled using the PacBio hierarchical genome assembly process (HGAP)/Quiver software package, followed by Minimus 2, and they were polished with Quiver (8). A single contig of 4,859,931 bp (G+C content, 52.17%) representing the complete chromosome and three contigs of 3,609 bp, 4,675 bp, and 221,009 bp representing 3 plasmids were generated. The largest plasmid is a resistance plasmid with incompatibility group A/C, and the best BLAST hit is *Aeromonas hydrophila* plasmid pR148 (GenBank accession no. JX141473); however, the pR148 plasmid is much smaller in size (165,906 bp).

The sequences were annotated using the NCBI Prokaryotic Genomes Automatic Annotation Pipeline (PGAAP) (http://www.ncbi.nlm.nih.gov/genomes/static/Pipeline.html) and have been deposited at DDBJ/EMBL/GenBank. The five prophages from the genome of CFSAN001921, Gifsy 2, ST64B, ST104, Svf, and *Burkholderia* BcepMu, were identified using Phast analysis (9).

During sequencing, epigenetic modifications of each nucleotide position were measured as kinetic variations (KVs) in nucleotide incorporation rates, and methylase activities were deduced from the KV data (10). Five DNA methyltransferase recognition motifs were detected by SMRT sequencing, and the genes encoding the various motifs are shown in Table 1. The methylome of this isolate was analyzed and deposited in Rebase (http://tools.neb.com/~vincze/genomes/view.php?view_id=27754).

**TABLE 1:** Summary of methyltransferase identified in *S*. Typhimurium CFSAN001921

Assignment	Methyltransferase specificity^*a*^	Type
M.SenTFI	CAGAG	III
M.SenTFII	G**A**GNNNNNNR**T**AYG	I
M. SenTfiii	A**T**GC**A**T	II
M.SenTFIV	GATC	Orphan
SenTFV	GATC**A**G	IIG

a The methylated bases, all m6A, are indicated by a boldface A if they are on the strand shown or a boldface T if they are on the complementary strand.

### Nucleotide sequence accession numbers.

The complete genome and plasmid sequences for *Salmonella* serovar Typhimurium CFSAN001921 are available in GenBank under accession no. CP006048, CP006050, CP006051, and CP006052.
