# Complete Genome Sequence of a Multidrug-Resistant *Salmonella enterica* Serovar Typhimurium var. 5− Strain Isolated from Chicken Breast

**DOI:** 10.1128/genomeA.00294-14

**Published:** 2014-04-03

**Authors:** Maria Hoffmann, Tim Muruvanda, Marc W. Allard, Jonas Korlach, Richard J. Roberts, Ruth Timme, Justin Payne, Patrick F. McDermott, Peter Evans, Jianghong Meng, Eric W. Brown, Shaohua Zhao

**Affiliations:** Division of Animal and Food Microbiology, Office of Research, Center for Veterinary Medicine, U.S. Food and Drug Administration, Laurel, Maryland, USA; Department of Nutrition & Food Science and Joint Institute for Food Safety & Applied Nutrition, University of Maryland, College Park, Maryland, USA; Division of Microbiology, Office of Regulatory Science, Center for Food Safety and Nutrition, U.S. Food and Drug Administration, College Park, Maryland, USA; Pacific Biosciences, Menlo Park, California, USA; New England BioLabs, Inc., Ipswich, Massachusetts, USA

## AUTHOR CORRECTION

Volume 1, no. 6, e01068-13, 2013. Page 2: The following should be added to the Acknowledgments. “Research reported in this publication was supported by the Small Business Innovation Research Program (NIGMS) of the National Institutes of Health under award number R44GM105125 to R.J.R.

The content is solely the responsibility of the authors and does not necessarily represent the official views of the National Institutes of Health.”

